# Supplementary material for: Kinetic Aspects of Ethylene Glycol Degradation Using UV-C Activated Hydrogen Peroxide (H2O2/UV-C)
Source: Molecules. 2024 Dec 26;30(1):49. doi: 10.3390/molecules30010049 (PMC11720823; doi:10.3390/molecules30010049)
Supplement: Supplementary file 1 [file molecules-30-00049-s001.zip › molecules-3345382-supplementary.pdf]

molecules

Article

Kinetic Aspects of Ethylene Glycol Degradation Using UV-C Activated Hydrogen Peroxide (H_2O_2/UV-C)

Timur Fazliev [1,2], Mikhail Lyulyukin [1,2], Denis Kozlov [1,2] and Dmitry Selishchev [1,2,*]

[1] Research and Educational Center "Institute of Chemical Technologies", Novosibirsk State University; Pirogova St. 2, Novosibirsk 630090, Russia; t.fazliev@g.nsu.ru (T.F.); m.lyulyukin@g.nsu.ru (M.L.); d.kozlov@g.nsu.ru (D.K.)
[2] Competence Center of the National Technological Initiative "Hydrogen as the basis of a low-carbon economy", Lavrentieva Ave. 7, Novosibirsk 630090, Russia
* Correspondence: d.selishchev@g.nsu.ru (D.S.)

Supporting information

1. Effect of light source on the temperature of reaction solution

All experiments were carried out in a thermal insulated glass reactor to maximize the utilization of total energy provided by the light source. To estimate the difference between processes with or without air purging, the temperature of reaction solution was measured during several experiments under different conditions (Figure S1). The reaction solution without air purging (i.e., H_2O_2/UV-C) heated more rapidly than in the case of H_2O_2 + air/UV-C that confirms a solution cooling by air flow. At the same time, the reaction solution with air flow, but without addition of H_2O_2 (i.e., air/UV-C) heated slower than in the case of H_2O_2 + air/UV-C. This result confirms the exothermicity of the ethylene glycol oxidation and its contribution to heating of the reaction solution.

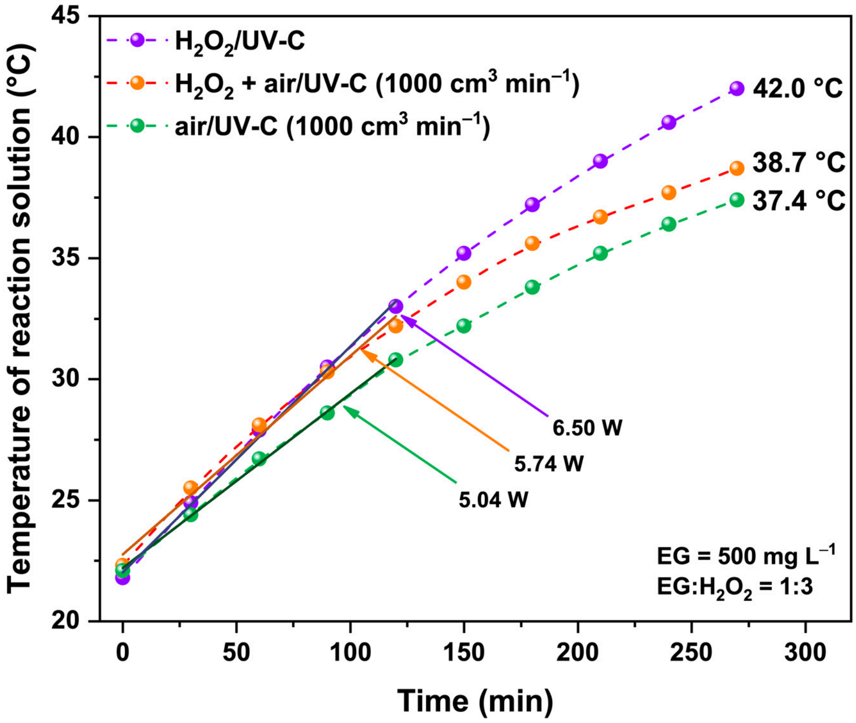

Figure S1. Changes in the temperature of the reaction medium under different conditions.

2. Products of the EG oxidation during UV-C treatment

The reduction of COD during the UV-C irradiation with the rate of 36 mg L^{-1} h^{-1} was noticed. This fact allows us to conclude that partial oxidation of EG in this solution occurs. To determine the main oxidation products, the reaction solution was analyzed via HPLC. According to the results shown in Figure S2, the EG concentration reduces from 8.05 mM to 6.51 mM after 3 h of irradiation (Figure S2a). The main products of EG photolysis are glycolaldehyde, acetaldehyde, formaldehyde, and formic acid (Figure S2b). The results obtained correlate well with the literature data [62,63,52]. Aldehydes can be readily formed during the VUV irradiation of the ethylene glycol solution even in deoxygenated medium because of dehydrogenation. Formation of acetaldehyde can be explained by the dehydration of the HO-•CH-CH_2-OH radical [52]. The C-C bond cleavage results in forming formaldehyde and formic acid. It can be concluded from the differences in EG kinetics that the formation of organic radicals without H_2O_2 occurs with a low rate. The presence of H_2O_2 and consequently the formation of OH• radicals lead to a greater rate of EG and byproducts oxidation.

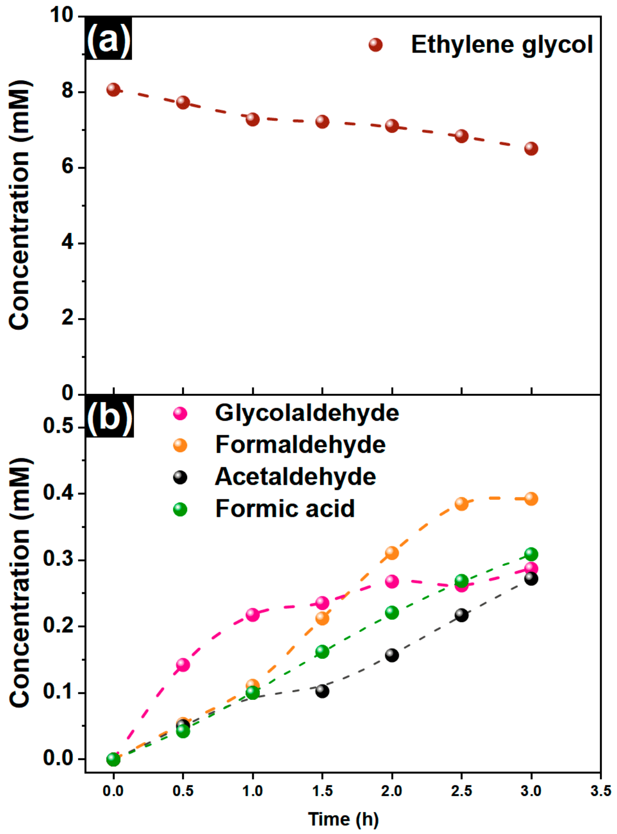

Figure S2. Concentration profiles of (a) EG and (b) oxidation products formed during the treatment of the EG solution with UV-C.

3. Mechanism of EG oxidation by H_2O_2

As found in this study, the presence of H_2O_2 during the treatment of EG aqueous solution using UV-C irradiation results in greater oxidation rate. Such an effect is a consequence of the formation of OH• radicals during H_2O_2 photolysis. These reactive oxygen species may be scavenged by EG triggering a formation of organic radicals and their further recombination and transformation (Figure S3). Thus, glycol aldehyde can be expected as a very first product of the EG oxidation in the H_2O_2/UV-C system. Its further transformations may result in glyoxal and glycolic acid depending on the carbon atom that is attacked by •OH radicals. However, it is difficult to predict a detailed mechanism of such radical transformations [55,56].

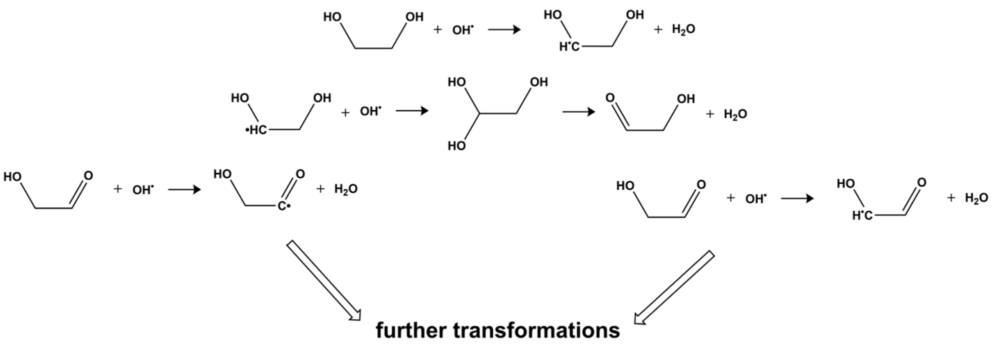

Figure S3. First steps of EG oxidation with OH• radicals as main reactive species.

4. HPLC chromatograms and retention times

Concentrations of EG and major organic intermediates, namely glycol aldehyde, formaldehyde, glyoxal, glycolic acid, glyoxylic acid, oxalic acid, and formic acid, were analyzed using high-performance liquid chromatography (HPLC). Details on HPLC equipment and operating parameters can be found in the section 3 (Materials and Methods) of the main manuscript. Figure S4 shows experimental chromatograms measured during the kinetic experiments. All identified components are marked. It is worth noting the presence of unidentified components in the chromatograms. They can be attributed to some esters of ethylene glycol or glycol aldehyde with formic acid. Due to the overlapping of glycol aldehyde/glycolic acid and formaldehyde/formic acid peaks, their concentrations were estimated using a system of linear equations with known molar coefficients in both PDA and RID detectors. Presence of such intermediates as formaldehyde, glycol aldehyde, glycolic acid, glyoxal, diethylene glycol, acetic acid, and formic acid was proved by GC-MS. At the same time, not all intermediates were identified due to their low concentrations and water, which significantly interferes with results of GC-MS analysis.

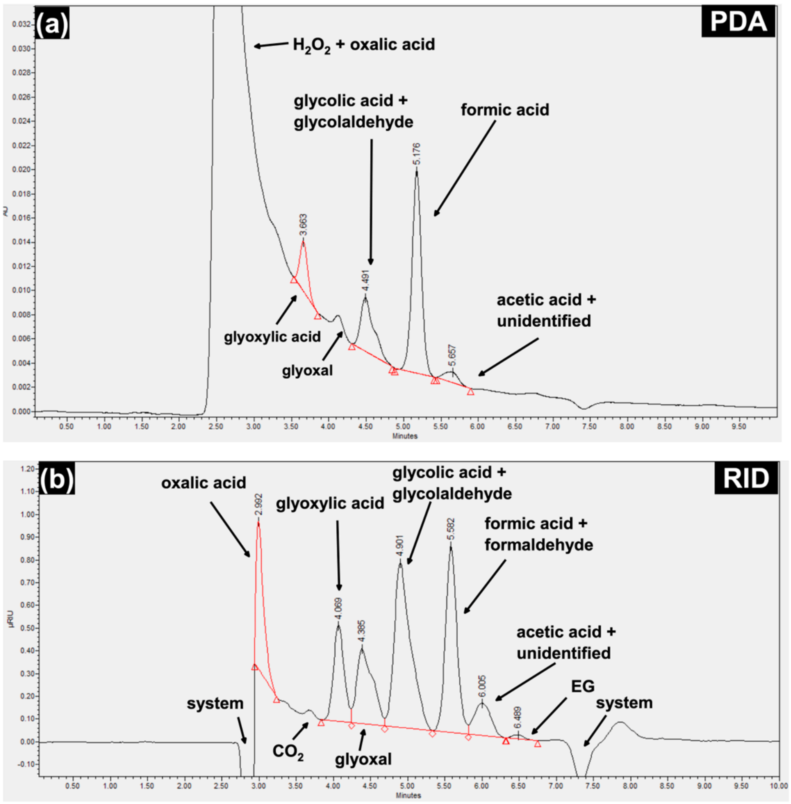

Figure S4. Experimental HPLC chromatograms of the reaction solution recorded via (a) photodiode array (PDA) and (b) refractive index detector (RID).

Retention times of corresponding components are listed in Table S1.

Table S1. Retention times of organic compounds detected in the reaction medium via HPLC.

Compound	Retention time in PDA [1] (min)	Retention time in RID [1] (min)
Oxalic acid	2.55	2.99
Glyoxylic acid	3.66	4.07
Glyoxal	4.06	4.37
Glycol aldehyde	4.50	4.90
Glycolic acid	4.62	5.02
Formic acid	5.18	5.58
Formaldehyde	– [2]	5.58
Ethylene glycol	– [2]	6.49

[1] retention times for PDA and RID detectors are different because these detectors are connected sequentially;
[2] formaldehyde and EG do not absorb at 210 nm.

5. Kinetics of EG removal

Time dependence of EG concentration corresponds well to the kinetics of a first-order reaction. The inset in Figure S5 shows a linear approximation in –ln(C/C_0) vs. t coordinates. The calculated efficient rate constant (k) in the H_2O_2/UV-C system (EG:H_2O_2 = 1:3) is found to be 3.5 ± 0.1 h^{-1}.

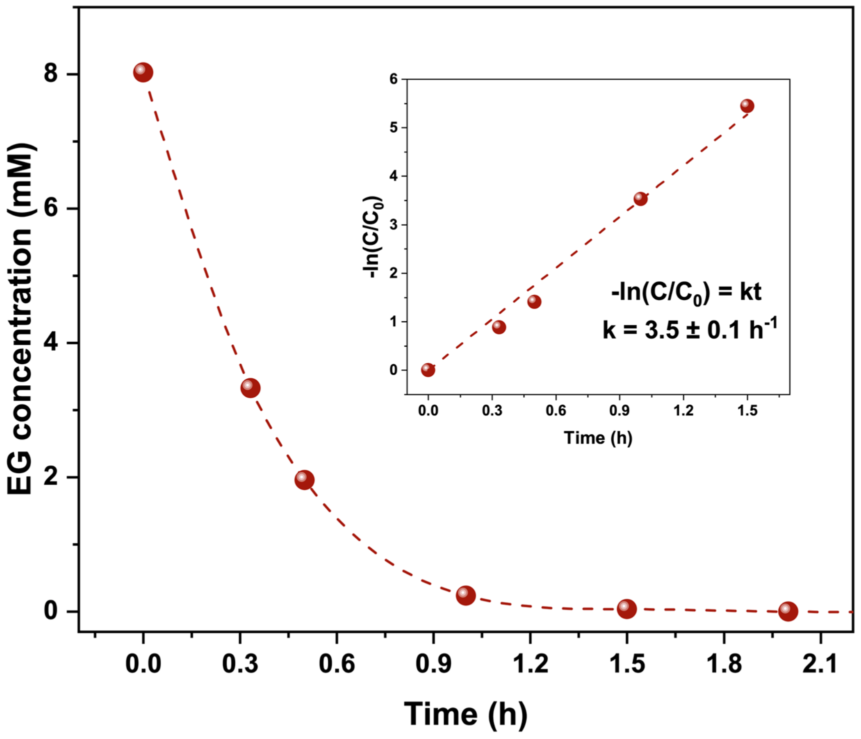

Figure S5. Concentration profile of EG removal and its linearization by corresponding coordinates.

6. Role of oxygen in the degradation mechanism

According to Vel Leitner and Dorè [54], oxygen in the H_2O_2 + air/UV-C system can boost oxidation of glycolic acid to glyoxylic and oxalic acids. Similar mechanism of glycolic aldehyde oxidation to formic acid can be proposed for the C-C bond cleavage pathway (Figure S6). At the first step, hydroxyl radical attacks glycolic aldehyde, thus leading to an organic radical. This radical can react with oxygen with the formation of peroxo

species, which can detach water and form glyoxal. Glyoxal can further react with H_2O_2 with the formation of formic acid [55,58].

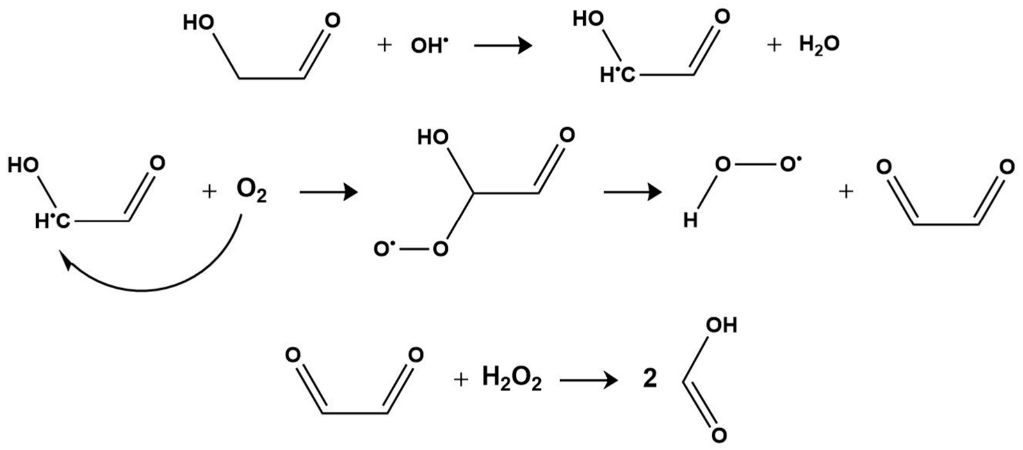

Figure S6. Proposed mechanism of oxidation though the C-C cleavage pathway boosted by oxygen.

7. Experimental setup

As stated in the Materials and Methods section, the kinetic experiments on EG degradation upon addition to H_2O_2 under activation with UV-C light were carried out in a 1.5 L glass vessel with an inner-placed quartz tube, equipped with a 10 W germicidal lamp. In some experiments, the reaction medium was purged with air or oxygen through the PTFE tube. The reaction medium was stirred magnetically during the experiments. Figure S7 shows a photograph of the experimental setup used in the study.

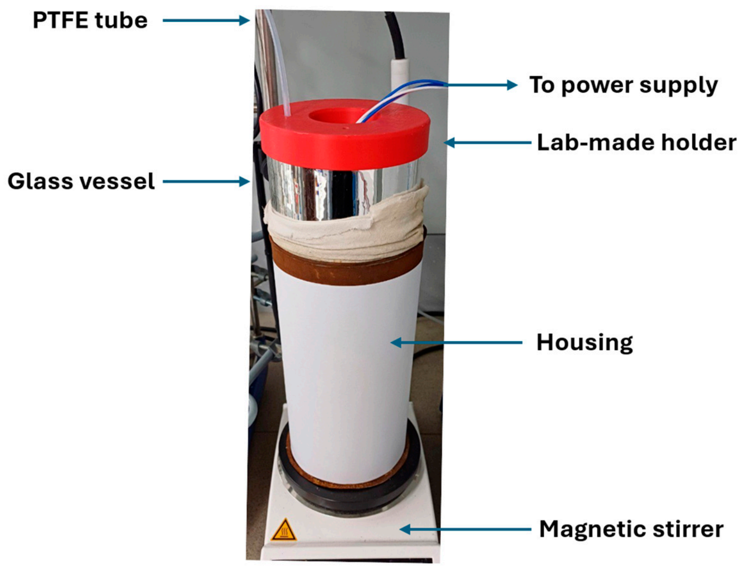

Figure S7. Photo of the experimental setup used for the EG degradation.
